# Supplementary figures and images for: A proteomic atlas of senescence-associated secretomes for aging biomarker development
Source: PLoS Biol. 2020 Jan 16;18(1):e3000599. doi: 10.1371/journal.pbio.3000599 (PMC6964821; doi:10.1371/journal.pbio.3000599)

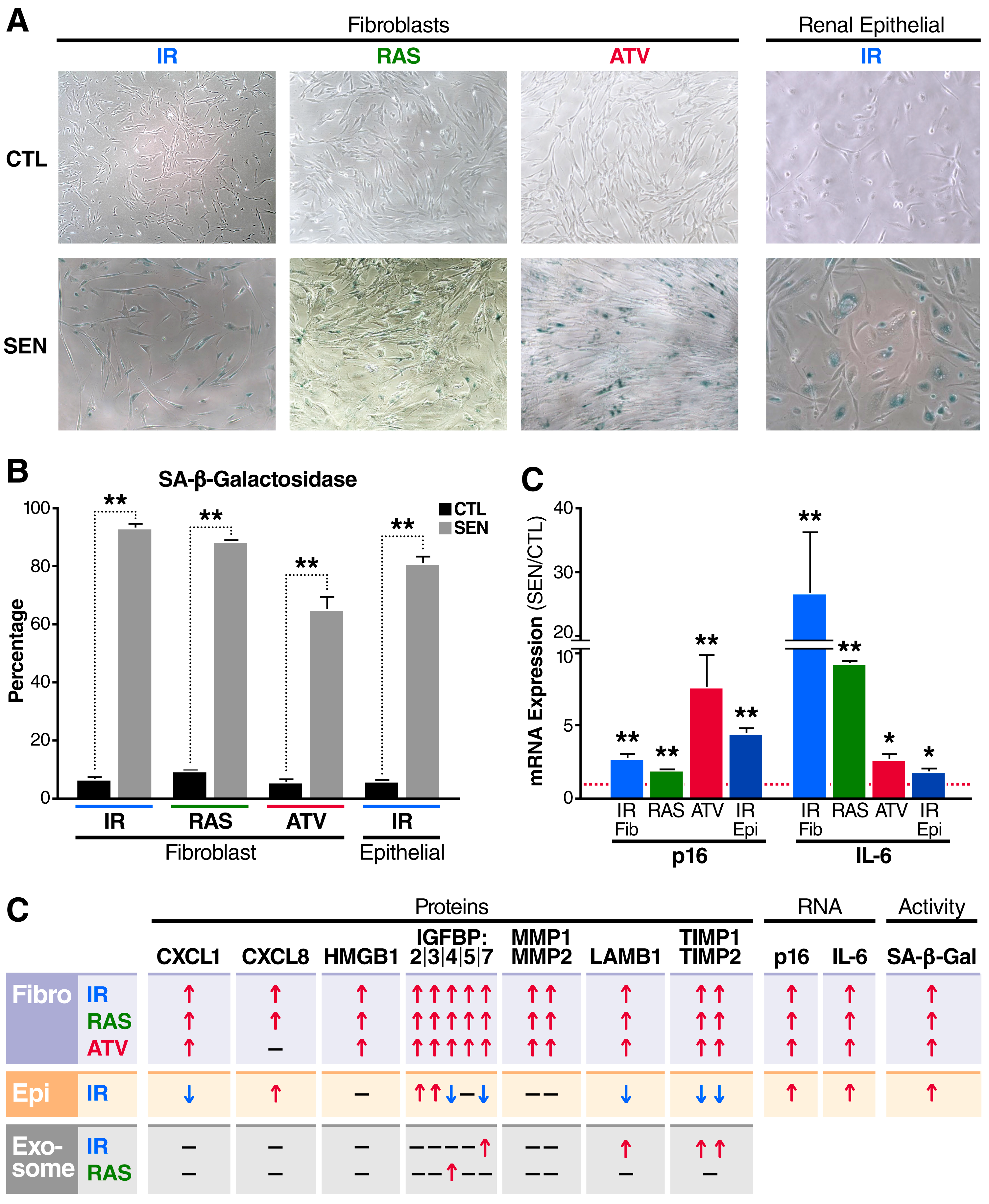

Supplement: S1 Fig — (A) Representative images of SA-β-Gal staining of senescent and control (quiescent) primary human lung fibroblasts and renal epithelial cells following induction of senescence by either IR, RAS, or ATV. (B) Quantification of SA-β-Gal–positive cells. (C) Levels of p16INK4a and IL-6 mRNAs determined by qPCR and expressed as fold change of senescent over control (red line) cells. (D) Commonly reported SASP factors for each inducer, cell type, and fraction. Red up-arrow = significantly increased (q-value < 0.05), blue down-arrow = significantly decreased (q-value < 0.05), hyphen (-) = not significant. ATV, atazanavir treatment; Epi, renal epithelial cell; Fib, fibroblast; IL-6, interleukin 6; IR, X-irradiation; qRT-PCR, quantitative real-time PCR; RAS, inducible RAS overexpression; SA-β-Gal, senescence-associated β-galactosidase; SASP, senescence-associated secretory phenotype. (TIF) [file pbio.3000599.s001.tif]

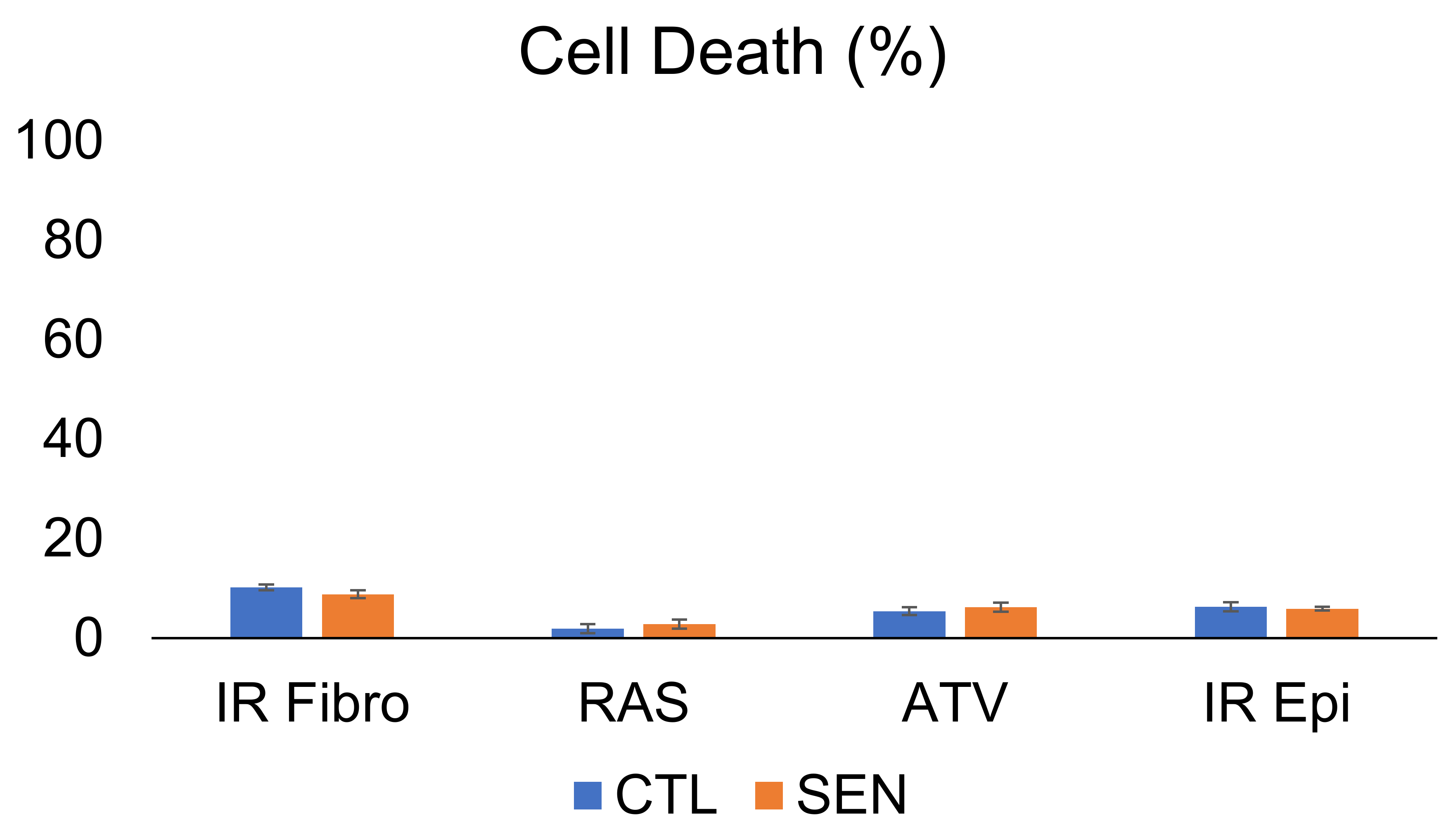

Supplement: S2 Fig — Amount of cell death over a 24-hour period as determined by Sytox Green viability dye assay or propidium iodide inclusion assay. (TIF) [file pbio.3000599.s002.tif]

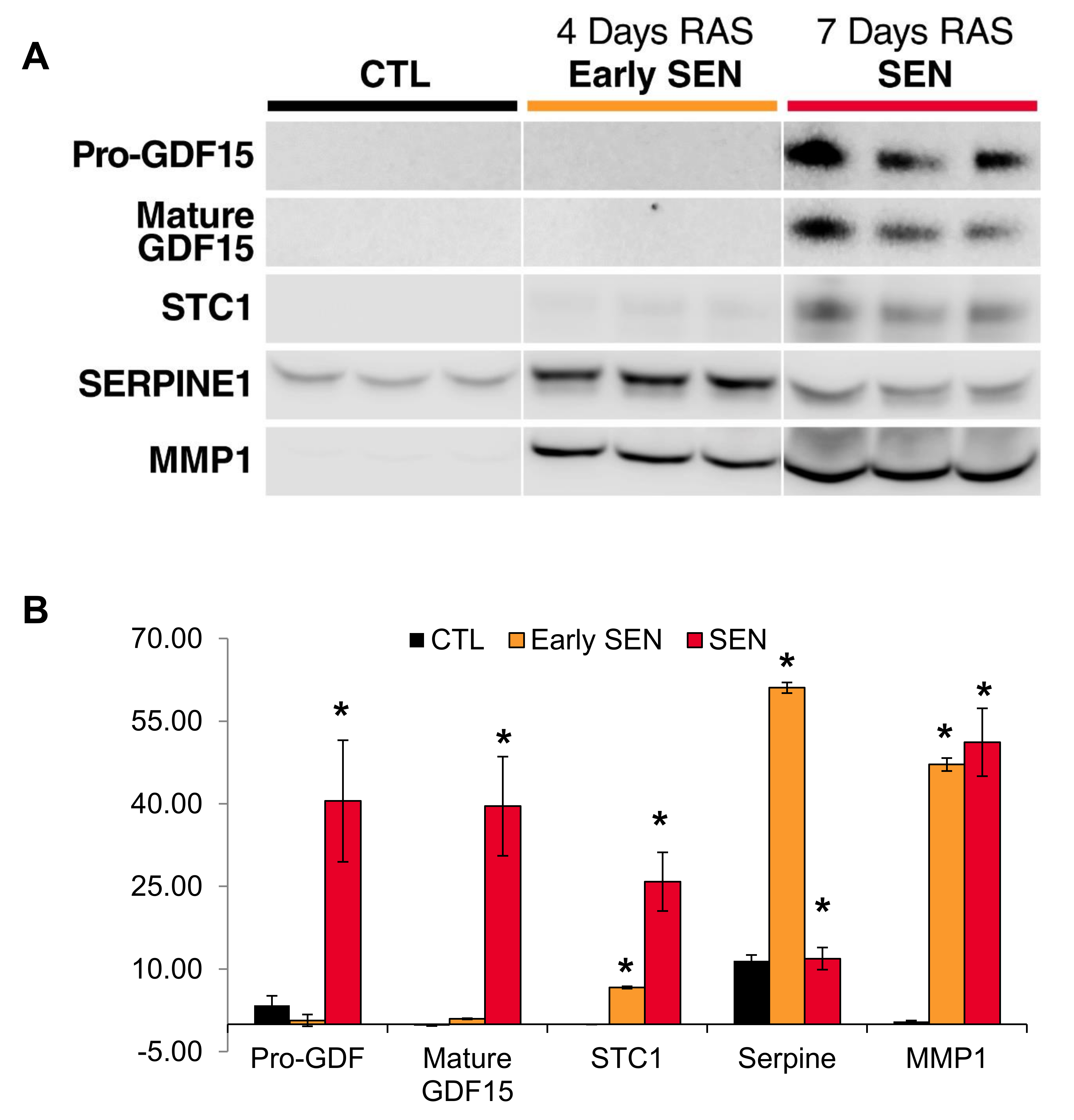

Supplement: S3 Fig — (A) Western blot exposures of top core SASP factors, GDF15, STC1, SERPINE1, and MMP1, in non-senescent control fibroblasts, early senescent fibroblasts (4 days of RAS induction), and fully senescent fibroblasts (7 days of RAS induction). (B) Densitometry analysis of western blot. *p-value < 0.05 versus CTL. CTL, quiescent control; GDF15, growth/differentiation factor 15; MMP1, matrix metalloproteinase-1; RAS, inducible RAS overexpression; SASP, senescence-associated secretory phenotype; SERPINE1, plasminogen activator inhibitor 1; STC1, stanniocalcin 1. (TIF) [file pbio.3000599.s003.tif]

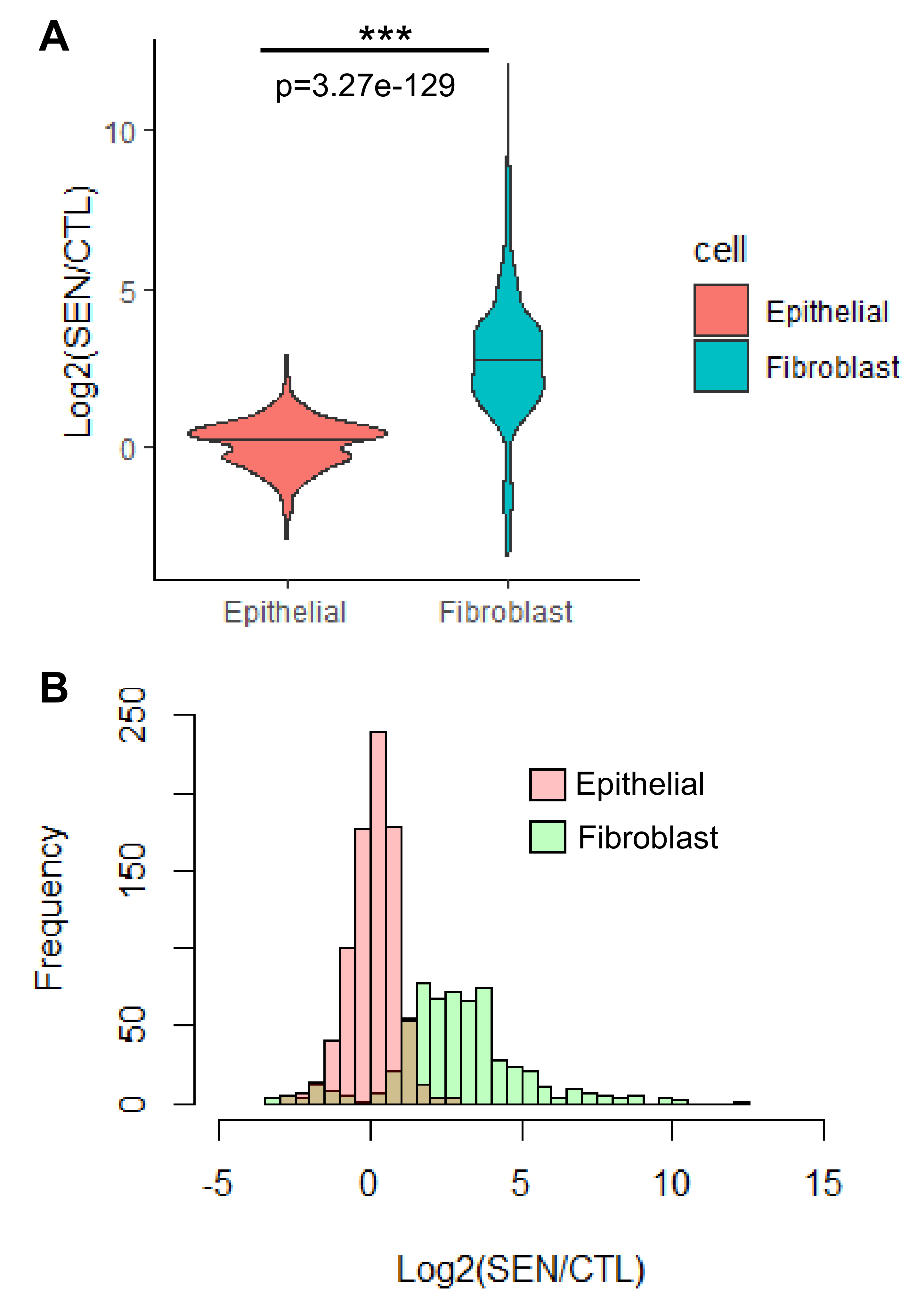

Supplement: S4 Fig — (A) Violin plot of all significant (q-value < 0.05) log2-fold changes in the sSASP of IR-induced fibroblasts and epithelial cells. (B) Histogram of all significant log2-fold changes in the sSASP of IR-induced fibroblasts and epithelial cells. IR, X-irradiation; sSASP, soluble senescence-associated secretory phenotype. (TIF) [file pbio.3000599.s004.tif]

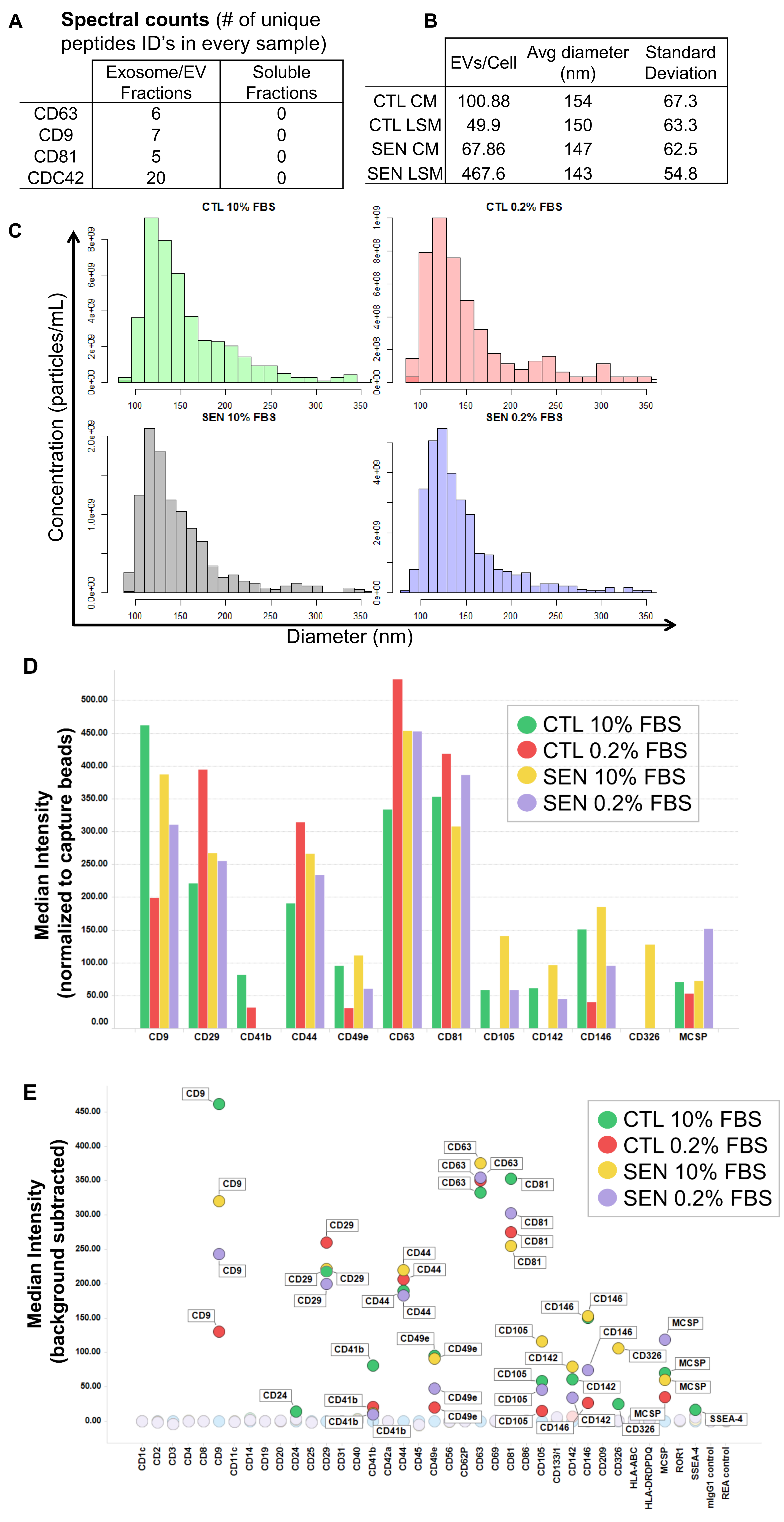

Supplement: S5 Fig — (A) Table of exosome- and EV-specific markers identified in exosome and soluble fractions of fibroblasts by mass spectrometry. Multiple peptides from defining exosome/EV markers were identified in the exosome fractions of RAS- and IR-induced senescence experiments, but none were detected in the soluble fractions. (B) Table showing EVs secreted per cell, average EV diameter, and standard deviation of EV diameter in senescent and control cells in complete (10% FBS) medium and low-serum (0.2% FBS) medium. The mean diameter of each condition is significantly different from each other condition (p-value < 0.00001, two-tailed t test). (C) Size distribution analysis of EVs secreted by senescent and control cells in complete and low-serum medium. (D) Exosome/EV-specific markers detected in isolated EV fractions in each treatment group, as measured by MACSPlex exosome detection kit. (E) Median levels of every surface marker measured in exosome/EV fractions by MACSPlex exosome detection kit. EV, extracellular vesicle; FBS, fetal bovine serum; IR, X-irradiation; RAS, RAS oncogene overexpression. (TIF) [file pbio.3000599.s005.tif]
