# Supplementary material for: A proteomic atlas of senescence-associated secretomes for aging biomarker development
Source: PLoS Biol. 2020 Jan 16;18(1):e3000599. doi: 10.1371/journal.pbio.3000599 (PMC6964821; doi:10.1371/journal.pbio.3000599)
Supplement: S1 Raw Images — (PDF) [file pbio.3000599.s015.pdf]

MMP1

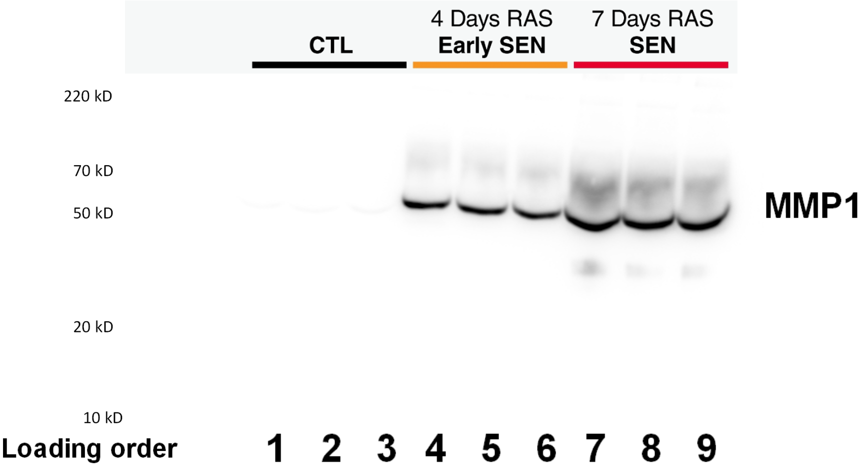

Azure c600 Imager
cSeries Capture Software
Chemi - Autoexposure

Fig S3A

GDF15

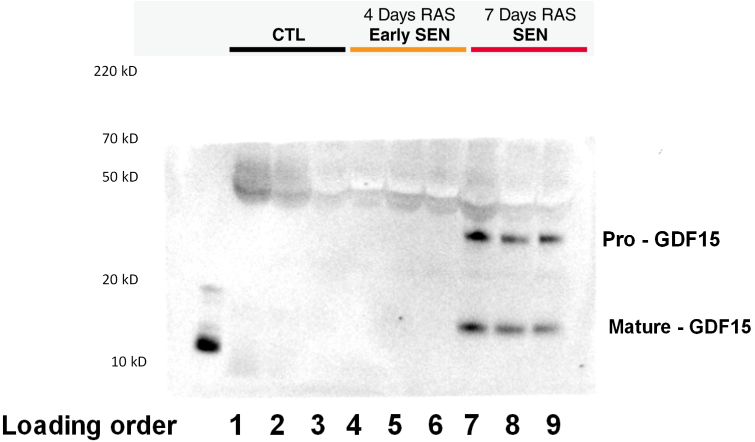

Azure c600 Imager
cSeries Capture Software
Chemi - Autoexposure

Fig S3A

SERPINE1

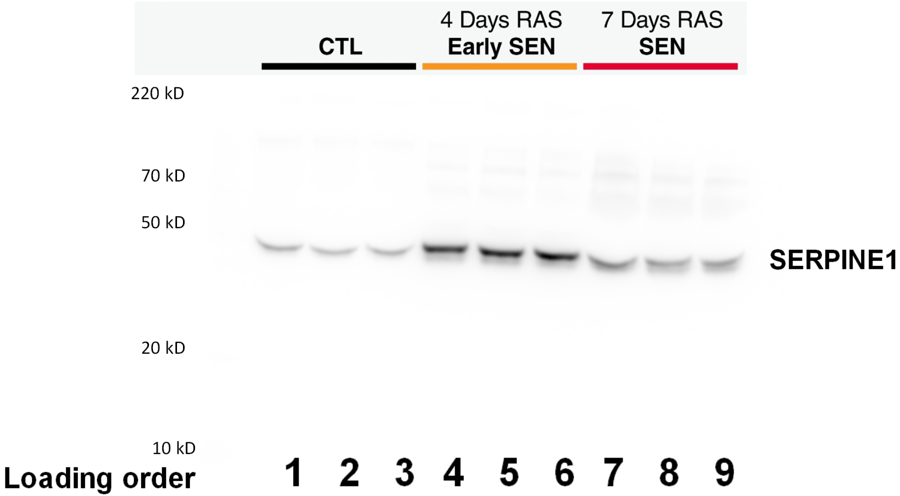

Azure c600 Imager
cSeries Capture Software
Chemi - Autoexposure

Fig S3A

STC1

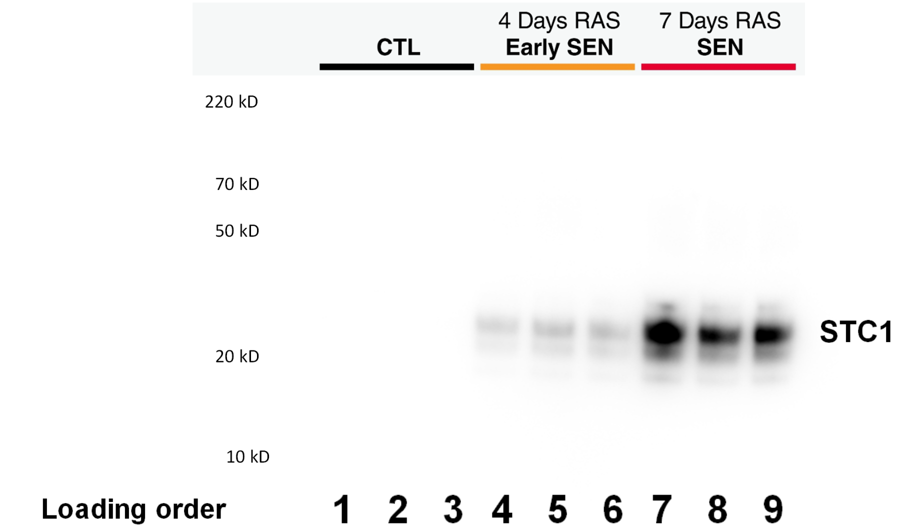

Azure c600 Imager
cSeries Capture Software
Chemi - Autoexposure

Fig S3A
