# Supplementary material for: A proteomic atlas of senescence-associated secretomes for aging biomarker development
Source: PLoS Biol. 2020 Jan 16;18(1):e3000599. doi: 10.1371/journal.pbio.3000599 (PMC6964821; doi:10.1371/journal.pbio.3000599)
Supplement: Attachment — Submitted filename: review_responses.docx [file pbio.3000599.s016.docx]

Dear Dr Schilling,

Thank you very much for submitting your manuscript "A Proteomic Atlas of Senescence-Associated Secretomes for Aging Biomarker Development" for consideration as a Methods and Resources article at PLOS Biology. Your manuscript has been evaluated by the PLOS Biology editors, an Academic Editor with relevant expertise, and by several independent reviewers.

In light of the reviews (below), we will not be able to accept the current version of the manuscript, but we would welcome resubmission of a revised version that takes into account the reviewers' comments. We ask that you address Reviewer 3's comments to the best of your ability but we will not insist on new proteomic data. As an additional request from the Academic Editor, please do also clarify whether the “plasma ageing markers” referred to in Fig. 5 (published by Tanaka et al.) are up- or down-regulated in the Tanaka signature, or remove the down-regulated markers from the analysis.

**Response**: We thank the academic editor for this suggestion and agree that the relevant plasma ageing markers should be increasing with age. We have removed all plasma markers that decrease with age from the analysis, including both Figure 5 (note: this is now Figure 6) and the underlying raw data in Table S4. All proteins except IGFBP3 were positively correlated with age from the “Core SASP”, which was removed from the analysis, and 9 proteins in total were negatively correlated from the larger “any inducer” comparison, which were also removed.

The corresponding results section of the text (page 17) and supplementary tables were also updated to reflect these changes.

Please note that we cannot make any decision about publication until we have seen the revised manuscript and your response to the reviewers' comments. Your revised manuscript is also likely to be sent for further evaluation by the reviewers.

……

You should also cite any additional relevant literature that has been published since the original submission and mention any additional citations in your response.

**Response**: We have added several relevant papers about GDF15. We have also added citations (below) or expanded our discussion as suggested by reviewers.

- Guo et al. “Senescence-Associated Tissue Microenvironment Promotes Colon Cancer Formation through the Secretory Factor GDF15”. 2019. *Aging Cell*
- Wiley et al. “SILAC Analysis Reveals Increased Secretion of Hemostasis-Related Factors by Senescent Cells.” 2019. *Cell Reports*
- Miroslava and Van Eyk. “Secreted Proteins as a Fundamental Source for Biomarker Discovery.” 2012. *Proteomics*.
- Capasso et al. “Changes in Autophagy, Proteasome Activity and Metabolism to Determine a Specific Signature for Acute and Chronic Senescent Mesenchymal Stromal Cells.” 2015. *Oncotarget*.
- Herranz et al. “MTOR Regulates MAPKAPK2 Translation to Control the Senescence-Associated Secretory Phenotype.” 2015. *Nature Cell Biology.*
- Marthandan et al. “Conserved Genes and Pathways in Primary Human Fibroblast Strains Undergoing Replicative and Radiation Induced Senescence.” 2016. *Biological Research*.
- Rai et al. “HIRA Orchestrates a Dynamic Chromatin Landscape in Senescence and Is Required for Suppression of Neoplasia.” 2014. Genes & Development.

Please note while forming your response, if your article is accepted, you may have the opportunity to make the peer review history publicly available. The record will include editor decision letters (with reviews) and your responses to reviewer comments. If eligible, we will contact you to opt in or out.

**Response**: We are happy to make the peer review history publicly available. If eligible we would like to opt in.

Before you revise your manuscript, please review the following PLOS policy and formatting requirements checklist PDF: http://journals.plos.org/plosbiology/s/file?id=9411/plos-biology-formatting-checklist.pdf. It is helpful if you format your revision according to our requirements - should your paper subsequently be accepted, this will save time at the acceptance stage.

**Response**: We formatted the manuscript according to the journal’s requirement.

Please note that as a condition of publication PLOS' data policy (http://journals.plos.org/plosbiology/s/data-availability) requires that you make available all data used to draw the conclusions arrived at in your manuscript. If you have not already done so, you must include any data used in your manuscript either in appropriate repositories, within the body of the manuscript, or as supporting information (N.B. this includes any numerical values that were used to generate graphs, histograms etc.). For an example see here: http://www.plosbiology.org/article/info%3Adoi%2F10.1371%2Fjournal.pbio.1001908#s5.

**Response**: We provided all numbers underlying any graphical displays in supplemental tables as requested in an excel sheet, Data S1, with each tab clearly labeled to correspond to a figure panel as shown in the example provided. We have also added additional cell culture information such as cell counts, size of culture flasks, and media volume as Table S6 and in the methods section.

For manuscripts submitted on or after 1st July 2019, we require the original, uncropped and minimally adjusted images supporting all blot and gel results reported in an article's figures or Supporting Information files. We will require these files before a manuscript can be accepted so please prepare them now, if you have not already uploaded them. Please carefully read our guidelines for how to prepare and upload this data: https://journals.plos.org/plosbiology/s/figures#loc-blot-and-gel-reporting-requirements.

**Response**: We provide all original western blot images in a combined PDF named ‘S1_raw_images.pdf’, as per the online instructions.

….

*****************************************************

REVIEWS:

Reviewer #1:

The paper is well written and is could be another step to identify a molecular signature for aging biomarkers. The effort to identify aging biomarkers that could be used as general markers for all aging-related conditions could be a chimera. Indeed, authors clearly evidenced many differences in the SASP contents of the senescent phenotypes they analyzed. Nevertheless, some markers may be present in many aging-related conditions and this paper gives indication about that. In my opinion, the efforts to identify common aging biomarkers should also consider that every senescent phenotype as a unique molecular profile (see for example: Oncotarget. 2015 Nov 24;6(37):39457-68. doi: 10.18632/oncotarget.6277. PMID: 26540573). I mean starting from differences could better lead to find common features. Authors should address this issue in the discussion.

1.1. **Response**: We completely agree that dissecting the differences in the molecular signature of senescent cells is as important as understanding common aspects of the SASP. As the reviewer points out, aging is likely a mixture of multiple different types of senescent cells and biomarkers may require multiple markers that cover different senescent cell types. In addition, specific diseases may be driven more by one or another senescent cell type or inducer, in which case we inducer-specific markers may be more relevant as biomarkers. We placed significantly more emphasis on this point in the updated manuscript, including new data analyses with figures (Fig 3, Table S7), and significant changes to the text.

To explore inducer-specific differences in senescent cells, we identified SASP components that are specific to senescence inducers, and that can be used as potential inducer-specific biomarkers. Based on published data on IR- and RAS-induced senescent fibroblasts and produced transcriptomic signatures of IR and RAS-induced senescence, we also generated combined transcriptomic signatures of senescent cells that are inducer-specific. In addition, we identified a signature of 5 proteins highly elevated in both the transcriptome and secretome of RAS-induced senescent fibroblasts that we propose may aid in identifying this subset of senescent cells *in vivo.*

The following changes were made to the text to better emphasize these points and address the reviewer’s comments.

- *[Page 11, line 210] “	We also generated proteomic sSASP signatures that were exclusive to two of the senescence inducers, IR and RAS (Fig 3A-B) to explore whether we could predict the originating inducer of senescence in published data. Due to the relative scarcity of published proteomic analyses of the sSASP, we compared our secretome analysis to published transcriptomic data. We prepared unique proteomic signatures of IR and RAS from our own data by filtering for secreted proteins, that were exclusively increased in the secretomes of either IR- or RAS-induced senescent fibroblasts (Table S7), and compared with published transcriptomic signatures of senescent cells for both stimuli [24,41–43]. The meta-transcriptomic profiles, as described by Hernandez-Segura et al. [24], are based on whole transcriptome (at least three replicates each) profiles of several fibroblast strains: MRC-5 and HFF datasets were used for analysis of IR-induced senescence and IMR90 were used for RAS-induced senescence. We combined these analyses with a more recent RNA-seq analysis of both IR-induced senescence in WI-38 and IMR-90 human fibroblasts and RAS-induced senescence in WI-38 fibroblasts [44], to generate lists of genes that are exclusively expressed in senescent fibroblasts following IR or RAS (Table S7). The combined transcriptome profiles contained 33 genes expression changes exclusive to IR-induced senescence and 1,749 genes expression changes exclusive to RAS-induced senescence. Likely due to the small overlap in IR-specific genes across transcriptomic studies, there was no overlap between IR-specific sSASP factors identified in our study and IR-specific transcriptome changes in senescent cells. However, of the 1,749 exclusively RAS-specific transcriptomic signature genes, we identified 26 proteins were exclusively secreted by RAS-induced senescent fibroblasts in this study (Figure 3C, Table S7), so we focused on generating a RAS-specific signature. The RAS signature included five proteins – CXCL5, MMP9, MMP3, CST4, and CCL3 – that were both robustly increased in both the secretomes (log2 fold change between 2.8 and 6.9) and transcriptomes (log2 fold-change between 4.9 and 9.1) (Fig 3D).”*
- [Page 23, line 476] *“It is important to note that while this study examines the SASP following several senescence-inducing stimuli, the originating stresses that drive an increasing senescent cell burden in vivo during aging and age-related diseases are unknown. We therefore focused our biomarker candidates on “core” soluble SASP components, which are presumably contributed by senescent cells originating from multiple stimuli. However, it is likely that this list of core components will decrease as additional stimuli and cell types are added to the SASP Atlas. Therefore, as senescent cells are better characterized in vivo, it will be important to tailor these biomarker candidates to better represent the originating cell types and stresses of senescent cells in the context of aging and specific diseases.*

*Inducer-specific biomarkers will be critical in cases where the originating stimuli is known, such as in treatment-induced senescence (i.e. patients taking Atazanivir or chemotherapeutics). Senescent cells also have a unique combination of changes in proteasome activity, autophagic flux and metabolic state depending on their originating stimuli, which have been proposed as inducer-specific biomarkers [73]. We also built inducer-specific senescence signatures based on our proteomic analysis and publicly available transcriptomic data (Fig 3, Table S7), and a combined signature for RAS-induced senescence. We propose these signatures will continue to evolve as other senescence-inducing stimuli are studied, and may serve as valuable biomarkers to identify the originating inducers of senescent cells in vivo. We will expand our efforts to build unique molecular profiles to distinguish between senescent cell types, or a subset of senescent cell types, in addition to pursuing a “core” signature.”*

--

Reviewer #2:

PLOS Biology

Title: A proteomic Atlas of Senescence-Associated Secretomes for Aging Biomarker development.

In this study, Basisty et al., show a comprehensive proteomic analysis on the components of the SASP using two different cell types and three different inducers of cellular senescence. After an exhaustive analysis, the authors determine that the composition of the SASP seems to be depending on the cell type as well as the type of inducer used to induce senescence. Also, the authors have found that senescent cells release more exosomes than control cells and that their protein composition differs from the secretory SASP (sSASP). Finally, by comparing their aSASP's data obtained in cells to the study using biomarkers in human plasma for diseases and aging, the authors conclude that a significant amount of SASP’s components can be considered as a good biomarker for aging and diseases.

Although this is a well written and very interesting work, I felt the authors could go a little more in deep about the meaning of their results., i.e., differences respect sSASP vs. exosomes, how they interpret the differences found in specific pathways in fibroblasts vs. epithelial cells or sSASP vs. exosomes, how the sSASP, that is intracellular phenomenon can be correlated to factors found in the plasma?

Overall, I found this work will significantly move the field forward with respect to cellular senescence and it links to biomarkers for aging and diseases.

2.1.	**Response**: We thank the reviewer for the feedback and added discussion points listed below regarding the differences between the soluble/exosome SASPs as well as inducer and cell-type differences.

With regard to the overlap with plasma proteins, we believe the SASP is a promising potential source of circulating factors. As reviewed by the Van Eyk group (Stastna et al. 2012. *Proteomics*), many groups found secreted proteins in abundance in plasma, therefore ‘secreted proteins can serve as a fundamental source for biomarker discovery’. In addition, our group recently showed that senescent cells, specifically, not only secrete bioactive molecules into the circulation, but that these senescence-associated circulating factors drive changes in hemostasis and blood clotting (Wiley et al. 2019. *Cell Reports*). We expanded our discussion on these points as well, as listed below.

- [Page 13 line 252] *“We compared the secretomes of radiation-induced senescent lung fibroblasts and similarly treated senescent renal epithelial cells to determine the cell-type specificity of the sSASP. For renal epithelial cells, the sSASP comprised a mixture of proteins with significantly lower or higher relative secretion (60% increased, 40% decreased), whereas 94% of protein changes in fibroblasts increased in secretion. The magnitude of the fold-changes in the sSASP were significantly higher in fibroblasts than in renal epithelial cells, regardless of inducer (Fig S5, p < 0.0001). For example, 531 of significant protein changes in the fibroblast sSASP were >2-fold, compared to 138 in the renal epithelial cell sSASP. However, for renal epithelial cells an additional 212 proteins showed significant changes between 1.5- and 2-fold increase or decrease.”*
- Please see **Response 1.1** **above**. Added discussion about inducer-specific differences starting on page 23, line 476.
- [page 18 line 347] “*Protein changes in exosomes released by senescent cells were also largely distinct from changes in soluble protein secretion obtained from matching cells. Of all 548 soluble proteins secreted at significantly higher levels in senescent fibroblasts following IR, only 51 (9.3%) significantly increased in the eSASP of senescent fibroblasts following IR, and 70 proteins (12.8%) significantly decreased in the eSASP. Accordingly, the pathways enriched within eSASP were largely distinct from the sSASP of irradiated fibroblasts. In addition to enrichment of proteins involved in membrane organization, such as cell adhesion and cell junction assembly proteins, the eSASP is uniquely enriched with signaling pathways such as RAS signaling, G-protein signaling, and prostaglandin synthesis and regulation (Fig 5C), as opposed to the metabolic, growth, and ECM-remodeling pathways found in the cell and inducer-matched sSASP. Full lists of proteins secreted by senescent exosomes are in Table S1.”*
- [page 24, line 486] *“Inducer-specific biomarkers will be critical in cases where the originating stimuli is known, such as in treatment-induced senescence (i.e. patients taking Atazanivir or chemotherapeutics). Senescent cells also have a unique combination of changes in proteasome activity, autophagic flux and metabolic state depending on their originating stimuli, which have been proposed as inducer-specific biomarkers [73]. We also built inducer-specific senescence signatures based on our proteomic analysis and publicly available transcriptomic data (Fig 3, Table S7), and a combined signature for RAS-induced senescence. We propose these signatures will continue to evolve as other senescence-inducing stimuli are studied, and may serve as valuable biomarkers to identify the originating inducers of senescent cells in vivo. We will expand our efforts to build unique molecular profiles to distinguish between senescent cell types, or a subset of senescent cell types, in addition to pursuing a “core” signature.”*
- [page 24 line 498] *“Surprisingly, many classical features associated with the sSASP, some of which were originally described in cultured fibroblasts, were not present or even changed oppositely in renal epithelial cells. Of note were an absence or reduced secretion of DAMPS, including HMGB1 and Calreticulin, danger signals that initiate inflammatory responses and immune clearance at sites of tissue stress and damage. Epithelial cells also showed significantly reduced secretion of proteins in pathways related to tissue structure and organization, that were significantly increased in fibroblasts, including ECM organization, focal adhesion and other pathways enriched with ECM proteins, proteases, and protease inhibitors. These differences between lung fibroblasts and renal epithelial cells highlight the importance of cell type origin on the heterogenous senescent phenotype and suggest that the SASP is individualized to cell type. Further characterization of the SASP from both fibroblasts and epithelial cells originating in other tissues will be needed to determine whether these cell-type differences in the SASP are specific to epithelial cells from the kidney or are generalized to other types of epithelial cells.”*
- [page 25 line 513] *“Our quantitative unbiased proteomic analysis of senescent fibroblasts and epithelial cells reveals a much larger and diverse SASP than initially reported. These SASP profiles contribute a number of new potential senescence, aging and disease biomarkers. By virtue of this proteomic analysis of secreted proteins, SASP profiles are also ideal candidates for plasma-based biomarkers, which are enriched among secreted proteins [74]. Senescent cells were recently shown to modulate hemostasis and clotting phenotypes in plasma in vivo [18], demonstrating that senescent cells secrete bioactive factors into circulation in vivo. The use of SASP factors as biomarker candidates is further supported by our analysis, which has indicated that core SASP factors are enriched among plasma biomarkers of aging in humans. In addition to general senescence biomarkers, many proteins will likely be specific to cell-type and originating stimulus.”*

Minor comments

1.	It called my attention that the authors used 10% CO2, there is a specific reason for that.

2.2.	**Response**: For culturing our cell lines, these are the standard gas conditions recommended by the supplier of the cell culture medium (DMEM). DMEM contains 3.7 g/L of H_2_CO_3_ as a buffer and requires this level of CO_2_ to maintain physiological pH in cell culture.

2.	What would be the importance of having two different compartments (cytosolic) and extracellular vesicles containing SASP components? Which one will be more relevant as a biomarker for aging and diseases?

2.3.	**Response**: This is an important question, and we believe it will be relevant to explore both soluble and exosome proteins in order to maximize our repertoire of potential biomarkers. Secreted proteins have been described extensively as a rich source of plasma and other biofluid biomarker candidates, particularly in cancer studies (Stastna et al. 2012. *Proteomics*). Exosomes have more recently emerged as another potential source of secreted biomarkers and have an added advantage (over soluble factors) that they may in some cases be traced back to their tissue of origin (Belove et al. 2016. *J Extracell Vesicles*). Extracellular vesicles also have a lower dynamic range than soluble plasma proteins, and therefore proteomic analysis is not as limited in the identification of low abundance proteins (Whitham and Febbraio. 2018. *Proteomics*).

Both the soluble and exosome SASPs are biologically active and important for senescence biology. The soluble SASP has been linked to many diseases, and extracellular vesicles secreted by senescent cells have been described to have roles in cancer progression (Lehmann et al. 2008. *Cancer Res*), in signaling and the propagation of secondary senescence (Borghesan et al. 2019. *Cell Reports)* and as markers of osteoarthritis (Jeon et al. 2019. *JCI Insight*).

We added the following discussion with regards to these points:

- [page 6 line 90] *“Exosomes secreted by senescent cells have been shown to have pro-tumorigenic effects [28], are associated with osteoarthritis [29] , and have the ability to induce paracrine senescence [26]. A previous proteomic analysis of protein cargo from senescent extracellular vesicles identified few known SASP factors [26], meriting further direct proteomic comparisons between extracellular vesicles (EVs) and soluble SASP factors.”*
- [page 17 line 336] *“The protein content of exosomes/EVs released by IR- vs RAS-induced senescent fibroblasts was largely distinct, sharing only 9 significantly altered proteins (Fig 5A). Exosomes/EVs were reported to contain protein signatures of their originating cells [28,47], offering a unique opportunity to identify senescence biomarkers with a degree of cell type specificity. Thus, exosome/EV proteins might distinguish senescent cells of different origins or resulting from different stressors. The membranes of exosomes are also representative of the originating cells [28,47]. Indeed, about 30% of all the exosome/EV proteins that increased upon senescence are plasma membrane proteins (Fig 5B), suggesting that exosomes/EVs might also identify cell type origins through their cell-surface proteins, although this will need to be experimentally confirmed.*

*Protein changes in exosomes released by senescent cells were also largely distinct from changes in soluble protein secretion obtained from matching cells. Of all 548 soluble proteins secreted at significantly higher levels in senescent fibroblasts following IR, only 51 (9.3%) significantly increased in the eSASP of senescent fibroblasts following IR, and 70 proteins (12.8%) significantly decreased in the eSASP. Accordingly, the pathways enriched within eSASP were largely distinct from the sSASP of irradiated fibroblasts. In addition to enrichment of proteins involved in membrane organization, such as cell adhesion and cell junction assembly proteins, the eSASP is uniquely enriched with signaling pathways such as RAS signaling, G-protein signaling, and prostaglandin synthesis and regulation (Fig 5C), as opposed to the metabolic, growth, and ECM-remodeling pathways found in the cell and inducer-matched sSASP. Full lists of proteins secreted by senescent exosomes are in Table S1.”*

3.	Interestingly miRNA has been involved as an important cargo of exosomes, however, the authors didn't describe if miRNAs were part of the exosomes' analysis. Did they analyze this?

2.4.	**Response**: This is an excellent point. miRNA cargo is an important aspect of exosomes and has been the focus of much of the research into exosomal cargo. We are interested in performing this analysis but have not done so as part of this study. As we are focused on proteomic changes, characterization of miRNA changes was outside of the scope of this particular study. We will investigate this question in future studies.

4.	Why the exosomes released by senescent cells induce either by IR or RAS have such a different proteomic composition? On the same experiment, is no clear whether the pathway analysis (Figures B and C) are the analysis on one type of stressor or whether it is an average between data from IR and RAS.

2.5.	**Response**: Like with the soluble SASP, we think most of the proteomic changes are likely due to the inducer. However, the exact mechanism is not clear. We hope to explore the mechanisms behind the secretome changes associated with each inducer in future studies.

For figure 5 (note: this was figure 4 in the previous version), panel B is showing the gene ontology compartment enrichment from exosomes released by senescent cells from either inducer (IR or RAS). Panel B is a pathway enrichment from only IR-induced senescence. We have edited the figure to clearly label this by adding titles to the panels and further clarifying details in the figure legends. We thank the reviewer for bringing it to our attention.

5.	Using the K means clustering, the authors suggested 3 proteins can be used as a surrogate biomarker of sSASP (lines 218 and 219). However, one of them, CXCL1, in epithelial cells has an opposite change with what was found in fibroblasts. Therefore, this is indicating that these 3 proteins can be used as biomarkers of the SASP for human fibroblasts, and maybe only the IMR-90. Wandering whether each cell type will have their own set of proteins that will serve as a biomarker of SASP to that type of cell.

2.6.	**Response**: Yes, we agree that these significant cell-type differences are critical to consider in developing senescence biomarker panels. Due to the many cell- and inducer-specific differences in the SASPs, we believe that senescence biomarkers will require panels of proteins that cover multiple possible cell types and inducers, as well as panels to identify cell-type and inducer specific senescent cells. We significantly expanded our emphasis of this important point in the revised manuscript, including a new analysis, figure (Fig 3 and Table S7), and several additions to the text, listed below:

- **Please see response 2.1 above**. Discussion of inducer-specific biomarkers begins on page 24, line 486.
- **Please see response 2.1 above**. Discussion of cell-type differences begins on page 24, line 498.
- **Please see response 2.1 above**. Discussion of secreted SASP proteins as biomarkers begins on page 25, line 513.

6.	What would be the hypothetical mechanism that allows sSASP proteins (secreted intracellularly) be also found in plasma?

2.7.	**Response**: Plasma is extremely rich in soluble protein factors and exosomes, most of which originate from secreted proteins, and a small fraction of which may be derived from leaking or dead cells. Presumably, proteins secreted from cells can be secreted directly into circulation, although the mechanisms by which proteins enter circulation once outside the cell are not fully known. However, it has been shown that secreted proteins are enriched with plasma biomarkers, making the characterization of secretomes a widely pursued method of identifying potential biomarkers (Stastna et al. 2012. *Proteomics*). In a recent study, our group has also demonstrated that senescent cells secrete bioactive factors into circulation that mediate hemostasis and blood clotting phenotypes, and strongly supports the presence of SASP proteins in plasma (Wiley et al.2019. *Cell Reports*). We have added discussion and references to the text:

- **Please see response 2.1 above**. Discussion of secreted SASP proteins as biomarkers begins on page 25, line 513.

Reviewer #3:

In this article, Basisty et al describe the development of a “proteomic atlas” of senescence-associated secretory pheotypes, which is currently online at www.saspatlas.com. The authors state that current knowledge of SASP factors is limited, due mainly to the array-based and transcriptional-based approaches used to detect soluble SASP factors. Correspondingly, they hypothesize that the SASP is more specific to source and condition than previously appreciated. Thus, the authors aim to stock their proteomic database with SASP factors derived from multiple senescence inducers and cell types, as measured using an unbiased mass spectrometry approach. They begin by inducing cellular senescence via RAS ovexpression, ATV treatment, or IR in primary human lung fibroblasts or primary human renal epithelial cells in vitro. The authors then utilize mass spectrometry to quantify corresponding proteomic changes in secreted soluble factors and exosomal content in comparison to control

cells. They find that induction of senescence leads to an overall increase in levels of SASP factors independent of induction method. However, they determine that classical SASP proteins respond differently in epithelial cells compared to fibroblasts, with many classical SASP proteins unchanged or even decreased in epithelial cells. Furthermore, when comparing the SASP induced by IR, RAS, or ATV treatment in fibroblasts, the authors find only a limited number of conserved changes (termed core senescence factors) with the majority exhibiting heterogeneous changes. Finally, the authors analyze exosomal SASP, and find that IR and RAS induce distinct proteomic signatures in eSASP.

Critique:

1.	At present, the data provided by the authors does not sufficiently address the gaps in knowledge that they lay out in the introduction. Specifically, the authors state three main aims: 1) “to develop robust and specific senescence and aging biomarkers” with “a comprehensive profile of the context-dependent and heterogenous SASP” (lines 60-61), 2) to build an “in-depth, quantitative and comparative assessment of SASPs originative from multiple stimuli and different cell types” (lines 87-88), and 3) to assess the “proteomic content and function of exosomes…secreted by senescent cells” (lines 91-92). As noted below, a number of issues arise.

•	Towards aim 1, the authors state that they are generating an “Atlas of senescence-associated secretomes for aging biomarker development.” To that end, they identify a list of >1000 genes that are “associated with senescence” due to their largely heterogeneous changes induced by IR, RAS, or ATX treatment in fibroblasts or epithelial cells. They then go on to note that 101/217 aging factors identified in Tanaka et al, 2018 overlap with this list, positing a connection between senescence secretomes and aging blood biomarkers. However, given the heterogeneous and large nature of the senescence secretomes they report, we take issue with the grouping of all proteomic changes into one list, termed new SASP. Additionally, given that this paper is framed in the context of aging, it is difficult to say how meaningful the new SASP is to physiological aging, given that IR, RAS-OE, and ATX treatment are not particularly physiological drivers of aging. To appropriately make these claims

or comparisons, it would be necessary, and of great interest, to add the secretome of senescent cells and non-senescent counterparts from an aged mouse or aged human to the database.

3.1.	**Response**: We thank the reviewer for a thorough feedback of our manuscript. We agree that the verbiage ‘new SASP, any inducer’ was not clear and have changed this to “SASP, any inducer” in the text and figure (Fig 6). (note: this was Fig 5 in the previous version of the manuscript)

We agree that adding senescent cells from aged mouse and human tissue would be the ideal, especially given our focus on aging biomarkers. Also, because it is currently not known which inducer of senescence is most physiologically relevant *in vivo* during aging. However, currently there are no methods to isolate senescent cells that arise *in vivo* from tissues (i.e., aged tissues), and we are limited to investigating senescence induced in culture*.* For these reasons, we examined multiple senescence inducers and cell types in culture and focus on the “core” markers as relevant to *in vivo* aging and have primarily focused on the overlap between the “core” (SASP factors present in all inducers) with plasma biomarkers of aging in humans. However, we also feel it will be critical to continue profiling SASPs following additional stresses and in additional cell types to eventually cover all possible SASP factors which might be seen *in vivo*.

Our future work will focus on adding secretomes from additional inducers and cell types to the SASP atlas to further define detailed SASP phenotypes. More importantly, we are developing approaches to isolate senescent cells from *in vivo*, which is a much-needed tool in the field.

We added significant discussion of these points to the text:

- **Please see response 2.1 above**. Discussion of inducer-specific biomarkers and *in vivo* relevance begins on page 23, line 476.
- **Please see response 2.1 above**. Discussion of secreted SASP proteins as *in vivo* biomarkers begins on page 25, line 513.

•	Towards aim 2, the authors fail to fully explore context specificity of the SASP, even within the limited scope of renal epithelial cells and lung fibroblasts. Given that the epithelial cell IR secretome shows major differences compared to the fibroblast IR secretome, it would be of great interest to test how the other senescence inducers affect the epithelial secretome.

3.2.	**Response**: This is a valid point and exploration of inducer effects in renal epithelial cells and other cell types would be of great interest, especially given the large cell-type and inducer dependence of the SASP phenotype thus far. We are continuing to explore the SASP in epithelial cells as well as other cell types using multiple inducers and will add these to the SASP Atlas as they are produced. We have expanded our discussion on the cell-type specific differences in the SASP in lung fibroblasts versus renal epithelial cells for the current manuscript and have further emphasized the importance of expanding the SASP atlas in multiple inducers and cell types both.

- **Please see response 1.1 above**. New inducer-specific analysis and results begins on page 11, line 210.
- **Please see response 2.1 above**. Updated cell-type differences results section begins on page 13, line 251.
- **Please see response 2.1 above**. Discussion of inducer-specific biomarkers and future outlook begins on page 23, line 475.
- **Please see response 2.1 above**. Discussion of cell-type differences and future outlook begins on page 24, line 637.

•	Towards Aim 3, the authors characterize the content of exosomes released by IR- vs RAS-induced senescent fibroblasts. Beyond listing the proteins present and comparing them, the authors do not conduct any informative analysis. They propose intriguing hypotheses that “exosome/EV proteins might distinguish senescent cells of different origins or resulting from different stressors” (lines 303-304); however, they do not assess whether any of the SASP factors might be predictive of cell source or senescent induction type. The authors should consider further analyzing their own dataset, or comparing it to published datasets (however limited by technical approach) to determine if such a stress/source signatures exist.

3.3.	**Response**: This is an interesting general point about using the proteomic profiles to create inducer- and cell-type specific biomarkers, and we agree this is potentially very valuable. Unfortunately, we are not able to do this analysis on exosomes due to the lack of published available datasets. However, we would like to do this in future studies as more data is made available either by other groups or by us. However, there are several transcriptomic studies on IR-induced and RAS-induced senescent fibroblasts with which we now started to compare our data. We have performed a new analysis, added figures (Fig 3, Table S7), and added corresponding text in the manuscript to describe and discuss these results.

To explore inducer-specific differences in senescent cells, we identified SASP components that are specific to senescence inducers to be used as potential inducer-specific biomarkers. We have also performed a meta-transcriptome analysis of senescent cells based on published data on IR- and RAS-induced senescent fibroblasts and produced transcriptomic signatures of IR and RAS-induced senescence. We additionally identified a signature of 5 proteins highly elevated in both the transcriptome and secretome of RAS-induced senescent fibroblasts, that we propose may aid in identifying this subset of senescent cells *in vivo.*

- **Please see response 1.1 above**. New inducer-specific analysis, results, and figures begins on page 11, line 210. Here we also compare with published datasets.
- **Please see response 1.1 above**. New discussion about core SASP and inducer-specific differences begins on page 23 line 476.
- **Please see response 2.1 above**. Discussion of inducer-specific biomarkers begins on page 24, line 486.
- **Please see response 2.1 above**. Discussion of cell-type differences begins on page 24, line 498.
- **Please see response 2.1 above**. Discussion of SASP as secreted biomarkers and *in vivo* relevance begins on page 25 line 513.

2.	The reasoning and details of the methodological approaches chosen by the authors are not clearly described.

•	It is unclear why the authors chose human lung fibroblasts and renal cortical epithelial cells for their studies. In the introduction, the authors mention that senescent cells are relevant to aging and disease. Are these cell types specifically relevant to those conditions?

3.4.	**Response**: With literally hundreds of cell types in mammals, our choice of cell types to interrogate is necessarily somewhat arbitrary. As for which senescent cell types are most relevant to diseases, particularly aging, *in vivo* is an excellent question and not entirely certain. Our choices were guided by the following factors:

Most studies of senescent cells *in vitro*, including transcriptomic and proteomic studies, have been conducted using lung primary lung fibroblasts, and thus many of the known senescent phenotypes including ‘pathological’ SASP factors emerged from studies conducted in these cells. We therefore use lung fibroblasts as a benchmark for characterizing senescent cells, as it is most comparable with previous work.

Senescence is also prominent in renal aging and chronic kidney diseases, as recently reviewed (Valentijin et al. 2018. J *Cell Commun Signal*). Senescent cells and SASP factors are increased in multiple kidney cell types during aging and disease and therefore potential biomarkers for kidney disease. In addition, elimination of senescent cells promotes healthy kidney functions. As potential plasma biomarkers, cells from the kidney are proximal to the blood and we reasoned that this may also make them relevant as potential sources of biomarkers.

In ongoing and future studies, we would like to expand our analyses into as many cell types as possible, including endothelial cells, adipocytes, and astrocytes.

•	For the detection of senescence-related secreted proteins, the authors profile conditioned media by mass spectrometry and use a cutoff value of q-value <0.05 and a 1.5-fold change (SEN/CTL). It is unclear if these values are somehow first normalized to cell number prior to comparing protein abundance. If not, it is impossible to determine whether the changes observed are due to altered secretions or artifacts related to differential cell abundance. This is of concern given that the clear majority of changes are observed in the same direction (upregulation). Additionally, if the images in figure S1A are representative, it appears cell density is not the same between CTL and SEN induced cells. These issues also apply to analysis of exosomal content.

3.5.	**Response**: All data was normalized to cells counts. We have added supplementary data (Table S6) containing the cell counts, culture flask, and volume of media used in all experiments, and have added this information to the methods section. The cells are all plated and senescence is induced at similar densities. However, the final cells count will sometimes vary depending on the inducer. For example, RAS-induction will cause a temporary growth acceleration prior to replication fork collapse and senescence, leading to higher final cell counts in RAS. Due to the evident variations in cell count between control and senescent cells, and sometimes between inducers, all data is normalized to cell counts.

The representative images in the SA-beta-galactosidase staining are not necessarily reflective of the original confluency of the cell culture. To perform SA-beta-galactosidase staining, senescent cells used in our experiments were trypsinized and an aliquot of cells were re-plated onto smaller plates prior to doing SA-beta-gal staining, which may not reflect the original cell density. We have added these details to the methods section and in Table S6.

- [Page 26 line 544] Under ‘Human Cell Culture and Primary Cell Lines’ subheading: *“Additional information about cell culture conditions, including seeding density, culture vessels, volume of medium, and final cell counts for each experiment are available in Table S6.”*
- [Page 30 line 636] Under ‘Senescence-Associated β-Galactosidase Staining’ subheading: *“Senescence-associated beta-galactosidase (SA-β-gal) activity was determined using the BioVision Senescence Detection Kit (Cat# K320-250). For each condition and replicate, cells were counted, and 7,000 cells were seeded into 8-well culture slides coated with poly-lysine (Corning #354632). After 24-hours, the SA-β-gal staining assay was performed as per the manufacturer’s protocol. For each experiment, approximately 100–150 cells were counted.”*
- [Page 32 line 680] Under ‘Processing, Quantification, and Statistical Analysis of MS Data’ subheading: “*To account for variation in cell number between experimental groups and biological replicates, quantitative analysis of each sample was normalized to cell number by applying a correction factor in the Spectronaut settings. Cell number and correction factors applied to each experiment are available in Table S6.*”

•	The methods are lacking in experimental detail. For example, they do not report the density at which cells were plated. Please ensure that all information needed to reproduce the results of this study are included in the methods.

3.6.	**Response**: **See previous response (3.5)**. We have normalized to cell counts and have added these numbers to the supplement (Table S6). We have also added additional information to the methods section regarding cell density.

•	Figure 1 suggests that senescence was induced in fibroblasts and epithelial cells by IR, RAS, ATV, prior to downstream proteomic analysis of secreted factors and exosomes. However, this is misleading as RAS overexpression or ATV treatment were only conducted on cultured fibroblasts, and not on epithelial cells. Similarly, exosomes were only collected from IR- or RAS-fibroblasts.

3.7.	**Response**: We agree with the reviewer and have removed the reference to renal epithelial cells from the figure.

3.	The text and figures are frequently vague, making it difficult for the reader to draw meaningful conclusions about the data being presented.

•	The authors state that (line 174) “the magnitude of the fold-changes in the sSASP were generally higher in fibroblasts than in renal epithelial cells, regardless of inducer” (2A). It is unclear where magnitude is represented in figure 2A. If it is the bold numbers with arrows, then the numbers in figure 2A are different than those in the text. To state that the magnitudes of the fold-changes in the sSASPs significantly higher in fibroblasts than epithelial cells, it needs to be supported by statistical approaches and concrete numbers. It is further unclear how the authors reach the conclusion that this change is independent of inducer, when epithelial cells only recieved IR. The authors should make sure to clearly explain what conditions/cell types, etc. were used in their analysis, and to accurately represent their results.

3.8.	**Response**: We agree that we should confirm this claim about the magnitude of fold-change differences in epithelial cells and fibroblasts by statistical methods and more descriptive figures. To address this, we have added a supplementary figure (Fig S4) that shows a violin plot and a histogram comparing the distribution of significant log2(SEN/CTL) changes in both cell types, along with p-value statistics. We have also specified in the text that the inducer-independent changes are referring only to fibroblasts.

- [Page 9 line 157] *“Most changes in the fibroblast sSASP, independent of inducer, exhibited increased secretion by senescent cells, with only 1-6% of proteins secreted at lower levels. In contrast, one-half to two-thirds of all significant protein changes in exosomes/EVs from senescent fibroblasts declined relative to quiescent cells (Fig 2A).”*
- [Page 13 line 252] *“We compared the secretomes of radiation-induced senescent lung fibroblasts and similarly treated senescent renal epithelial cells to determine the cell-type specificity of the sSASP. For renal epithelial cells, the sSASP comprised a mixture of proteins with significantly lower or higher relative secretion (60% increased, 40% decreased), whereas 94% of protein changes in fibroblasts increased in secretion. The magnitude of the fold-changes in the sSASP were significantly higher in fibroblasts than in renal epithelial cells, regardless of inducer (Fig S5, p < 0.0001). For example, 531 of significant protein changes in the fibroblast sSASP were >2-fold, compared to 138 in the renal epithelial cell sSASP. However, for renal epithelial cells an additional 212 proteins showed significant changes between 1.5- and 2-fold increase or decrease.”*

•	Throughout the entirety of the text, phrases such as “most proteins” (line 125), “large fraction” (line 146), and “a more even mix” (line 173) need to be replaced with specific numbers and statistics.

3.9.	**Response**: We thank the reviewer for the thorough read and have corrected the lines referenced with specific numbers. We have also read through the manuscript and changed other vague wording where possible.

•	The authors frequently round in the text (ex: “~1700”- line 145). In some cases, it represents improper rounding such as: “about 68 per cell and 49 per control cell” (line 294) rounded from 49.9. This raises concerns as to the rigor of the analysis.

3.10.	**Response**: We thank the reviewer for catching this mistake. We have corrected this rounding error and have double checked other numbers presented throughout the text for accuracy.

•	The authors confirm the presence of beta-galactosidase, p16, and IL-6 expression in each of their conditions (figure S1A-C). They then state that “there was no detectable cell death” (line 131, figure S2). First, this is not reflected in the data: Figure S2 shows viability at ~90% and cell death at ~5%. It is also unclear what cell type and condition is being assessed in figure S2. Cell viability should be reported for all models of senescence, as cell death could be a major contributor to the secretome assessed.

3.11.	**Response**: We have compiled data for cell viability for all conditions, including IR fibroblasts, RAS fibroblasts, ATV fibroblasts, and IR epithelial cells, as requested by the reviewer and included them all in the figure (Fig S2), with an updated figure legend and methods section specifying how cell viability was measured. Cell viability was measured by either SYTOX green or propidium iodide exclusion assays. We have also updated the methods section regarding how these assays were performed.

- [Page 30 line 627] Under ‘Cell Viability Assays’ subheading: *“Cell viability was assessed with SYTOX Green Nucleic Acid Stain (Invitrogen #S7020) or propidium iodide (Thermo #P1304MP) inclusion assay. Senescent and control cells were incubated for 24 hours in serum-free medium containing SYTOX Green with continuous imaging. Cell death was quantified by counting total SYTOX Green positive nuclei (523 nm) after 24-hours. For propidium iodide inclusion assays, cells were incubated with propidium iodide for 1 hour, and cell death was quantified by counting the number of propidium iodide positive cells (617 nm).”*

4.	The combinations of data employed for pathway analyses throughout the paper are confusing. The fact that the data were combined at all is principally in conflict authors’ idea that the SASP is cell type and senescence-induction specific. Moreover, the conclusions drawn from the pathway analyses presented throughout the paper are primarily speculation, given the lack of experimental data provided to support them.

3.12.	**Response**: In response to the reviewer’s comment, we have added additional discussion and analysis focusing on the cell and inducer-specific components of the SASP to give the paper more balanced focus on the “core” SASP, with features common to senescent fibroblasts under each condition, and inducer- and cell-type specific SASPs. The majority each SASP in the inducers and cells we tested are different from any other SASP, so it is true that they are largely heterogeneous. At the same time, a subset of the SASP from each inducer in fibroblasts are shared, which we call the ‘core’ SASP. We believe that there is value in exploring both the shared components of the SASP as well as the inducer- or cell-specific components of the SASP, and have added further discussion to clarify this in the text as well as new results on inducer-specific transcriptome and secretome signatures, as stated above, in response to reviewer 1 comment #1 (response 1.1).

We have made the following modifications to the results and discussion to the text to better clarify these points:

- **Please see response 1.1 above** for added discussion regarding the biological relevance of the “core” and inducer specific SASP as potential human biomarkers, starting on page 23 line 476.
- **Please see response 2.1 above** for added discussion regarding the cell-type specific differences, starting on page 24 line 498.
- **Please see response 2.1 above** for added discussion regarding the biological relevance of secreted SASP proteins as plasma biomarkers of aging and senescence in humans, starting on page 25 line 513.

•	The authors perform “pathway and network analysis of proteins increased in the sSASPs of [IR] fibroblasts and [IR] epithelial cells” and conclude that “many pathways were commonly enriched in both the epithelial and fibroblast sSASPs (Fig 3C and Fig 2C)” and that “pathways enriched uniquely by epithelial cells included protein translation and degradation.” These conclusions suggest that the authors did pathway and network analysis on the increased sSASPs from fibroblasts and epithelial cells separately, however this is not what is represented in figure 3C or 2C.

3.13.	**Response**: We thank the reviewer for bringing to our attention that it is unclear how these conclusions relate to the pathway analysis in Fig 3C and Fig 2C. The pathway analysis depicted in figure 3C and 3D was conducted on only renal epithelial cell SASP, on proteins that were secreted at significantly higher levels in senescent versus control (3C) or significantly lower levels in senescent versus control (3D) cells. In our discussion we were comparing these changes with fibroblasts at the level of pathways, rather than individual protein level. We have adjusted the figure legend to make this clearer. We have also added titles to the figure panels in both figures to make each clearer. Please note that Fig 3 referred to above is now Fig 4 in the revised manuscript.

- [Page 14 line 274] *“****Fig 4. Epithelial cells and fibroblasts exhibit distinct sSASPs.*** *(A) Number of proteins identified and significantly altered in the sSASP of irradiated fibroblasts and epithelial cells. (B) Venn diagram comparing proteins significantly increased in the sSASPs of senescent fibroblasts and epithelial cells, both induced by X-irradiation (q< 0.05). (C) Venn diagram comparing protein increases in the fibroblast sSASP vs decreases in the epithelial sSASP. (D) Pathway and network analysis of secreted proteins significantly increased in epithelial cell sSASP. (E) Pathway and network analysis of proteins significantly decreased in the epithelial cell sSASP.”*

•	The authors conclude, for example, that the “enrichment of neurodegeneration-associated proteins and pathways suggests that senescent cells contribute to neurodegenerative diseases” (lines 209-211). This conclusion is very misleading to the reader, as the authors 1) claim cell-type specificity but are working with fibroblasts and epithelial cells to draw conclusions about pathological, aged brain cells and 2) the authors conducted no experimental analysis to validate these findings, or even cross-reference their capacity to be “biomarkers” with data from published plasma biomarker studies.

3.14.	**Response**: We appreciate the reviewer’s comment on this figure and have decided to remove this figure panel and the accompanying text from the manuscript. Considering the reviewer’s comments, we would like to limit the scope of the discussion to fibroblast-related functions and pursue a more rigorous biological validation of these findings in relevant cell types or *in vivo* in future studies.

5. The database itself is an interesting idea, and the current interface is well-made. The graphics generated by the SASP query tool is intuitive and helpful. However, exporting the data is a bit difficult. Currently, downloading the data table generates an excel populated with what appears to be the logRatio – however this is not clearly labeled. It would be nice to have the downloaded table include the logRatio and -log10(qvalue).

3.15.	**Response**: We appreciate this feedback and have added the -log10(qvalue) column and relabeled the log2Ratio column in the downloadable table, as requested.

Minor.

•	Figure 4B: The percent label is vague. What is this a percentage of?

3.16.	**Response**: This is the percentage of all proteins identified in the exosome fractions. We have re-labeled the graph “% of all eSASP proteins”

•	Figure S4: Where is CD63, CD9, etc. values for CTL fractions?

3.17.	**Response**: The values in this panel represent spectral counts from both senescent and control fractions. For example, the 6 unique CD63 peptides identified values for IR (under exosome/EV fraction) were identified in all exosome samples from the IR experiment, including both senescent and control exosome fractions. This was not clear from the current labeling and we appreciate the comment. We have adjusted the table and the labels to indicate these values represent both senescent and control exosome fractions. (Note this is now Fig S5.)

•	Figure S4: The authors report CD81 and CDC42 values in figure S4, but do not discuss in the text.

3.18.	**Response**: We have added references to CD81 and CDC42 to the text (page 17 line 323).

•	Figure S4: The authors state that the mean diameter and size distribution of senescent and control exosomes/EVs were similar. Please provide statistics to show that they are not different.

3.19.	**Response**: We have added statistics and have found that the differences are significant, albeit small, as determined by a two-tailed t-test (page 17 line 332). We thank the reviewer for the comment. (Note this is now Fig S5.)

•	Figure S4: Graphs depicting the concentration and diameter of exosomes should use the same x-axis to better facilitate the comparison.

3.20.	**Response**: We have adjusted the graphs to be plotted on the same x-axis in Figure S5.

•	There are numerous grammatical errors. For example, “Several types of stress elicit a senescence and SASP response, which in turn can drive multiple phenotypes and pathologies associated with mammalian.” (line 63).

3.21.	**Response**: We thank the reviewer for bringing this to our attention. We have corrected the typo mentioned and conducted a thorough read through of the text for grammatical and spelling errors.

--

Reviewer #4:

This is an interesting and valuable report. It is essentially descriptive but the quality and extent of the analyses is relevant. Particularly the finding of common factors present in the SASP and in the plasma of old donors. I have a few comments that the authors may want to address:

We thank the reviewer for the encouraging words, and we have addressed the below comments.

1.	Perhaps is worth mentioning in the abstract that soluble and exosome SASPs have been analyzed.

4.1.	**Response**: We agree and thank the reviewer for pointing this out. We have added this into the abstract:

- Page 2 line 22: “Here, we present the ‘SASP Atlas’, a comprehensive proteomic database of soluble proteins and exosomal cargo SASP factors originating from multiple senescence inducers and cell types.”

2.	Regarding this sentence in the Introduction: “We also present the first comprehensive proteomic analysis of the exosomal SASP (eSASP), which is largely distinct from the sSASP”. Please avoid claims of priority and acknowledge (cite, compare and discuss) data in https://www.ncbi.nlm.nih.gov/pmc/articles/PMC6613042/ (incidentally this study identifies 1,600 proteins in the eSASP)1

4.2.	**Response**: We reworded the statement to remove priority claims and acknowledge the proteomic analysis previously performed by Borghesan et al. We have also added discussion which compares our findings with this previously described proteomic analysis.

- [Page 6 line 90] *“Exosomes secreted by senescent cells have been shown to have pro-tumorigenic effects [28], are associated with osteoarthritis [29] , and have the ability to induce paracrine senescence [26]. A previous proteomic analysis of protein cargo from senescent extracellular vesicles identified few known SASP factors [26], meriting further direct proteomic comparisons between extracellular vesicles (EVs) and soluble SASP factors.”*
- [Page 8 line 154] *“Notably, the protein cargo of exosomes/EVs released by senescent cells was distinct compared to that from non-senescent cells (Fig 2A), supporting the existence of an exosome/EV SASP (eSASP) in addition to the sSASP, as described [26]. Most changes in the fibroblast sSASP, independent of inducer, exhibited increased secretion by senescent cells, with only 1-6% of proteins secreted at lower levels. In contrast, one-half to two-thirds of all significant protein changes in exosomes/EVs from senescent fibroblasts declined relative to quiescent cells (Fig 2A).”*

2. Typos: Fig S1D is mislabeled as S1C, and it says LAMB1 instead of LMNB1.

4.3.	**Response**: We have corrected these mistakes and thank the reviewer for bringing them to our attention.

4.	The nomenclature is confusing: “secretome” or “SASP” sometimes refers exclusively to soluble SASP (sSASP) and not exosomes (for example Fig. 2A classifies “secretome” vs “exosomes”), however in other places both are considered SASP (sSASP vs eSASP). There are multiple instances in the text where the meaning has to be inferred from the context. Please, be consistent and simple.

4.4.	**Response**: We appreciate the feedback and have adjusted the text throughout to have more specific and consistent wording when referring to soluble versus exosome SASP, as well as the inducer of origin.

5.	Fig. 2A: please clarify the meaning of the numbers:

4.5.	**Response**: These numbers refer to the total number of proteins identified on the top of each box, and below this number is the number of proteins that were secreted at significantly higher levels (left with a red up arrow) or lower levels (right with a blue down arrow) by senescent cells versus control cells. We have altered the figure and legend for clarity. We have indicated which protein numbers refer to proteins that are identified and changed in the figure and added a legend for the arrows.

a.	The first and second columns, do they refer to proteins that change both in fibros and epi combined?

4.6.	**Response**: These are changes that occur in only fibroblasts following each inducer. We have changed the figure now to clarify what is represented with clear titles for each figure panel, and now feature only fibroblasts SASP in figure 2. We have moved the comparison with epithelial cells to Fig 4.

b.	The third column (labelled “fibro” and “epi”), does it refer to proteins that change with any of the three stressors combined? The fact that the numbers in “fibro” coincide exactly with the numbers in the secretome of IR-senescent cells, is it just a coincidence? Or a typo?

4.7.	**Response**: This portion of panel 2A panel was moved to Fig 4, which compares fibroblasts and epithelial cells, to avoid confusion and redundancy regarding the figure, which was intended to focus mostly on changes in fibroblasts. The numbers coincided for both because they were both the number for IR-induced senescent fibroblasts only. We moved this to avoid redundancy and focus the figure on fibroblasts.

6.	In line 332: “core SASP” (SASP components resulting from all senescence inducers). I would rather say: “core SASP” (SASP components common to all senescence inducers).

4.8.	**Response**: We agree and thank the reviewer for the clearer wording of this statement. We have adjusted as suggested.

--------------------
