# Supplementary material for: A proteomic atlas of senescence-associated secretomes for aging biomarker development
Source: PLoS Biol. 2020 Jan 16;18(1):e3000599. doi: 10.1371/journal.pbio.3000599 (PMC6964821; doi:10.1371/journal.pbio.3000599)
Supplement: Attachment — Submitted filename: review_responses_v2.docx [file pbio.3000599.s017.docx]

Dear Dr Schilling,

Thank you for submitting your revised Methods and Resources entitled "A Proteomic Atlas of Senescence-Associated Secretomes for Aging Biomarker Development" for publication in PLOS Biology. I have now obtained advice from the original reviewers and have discussed their comments with the Academic Editor. Based on the reviews, we will probably accept this manuscript for publication, assuming that you will correct the manuscript to address the remaining points raised by Reviewer 4 and carefully revise the entire manuscript to catch all such inconsistencies and errors. Please also make sure to address the data and other policy-related requests noted at the end of this email.

Response: We appreciate the editor’s comments. We have addressed all inconsistencies pointed out by the Reviewer 4, in addition to a few other minor corrections that we made upon a thorough re-reading of the manuscript, figures, and tables. We have followed the data and other policy-related requests by the journal.

We expect to receive your revised manuscript within two weeks. Your revisions should address the specific points made by each reviewer. In addition to the remaining revisions and before we will be able to formally accept your manuscript and consider it "in press", we also need to ensure that your article conforms to our guidelines. A member of our team will be in touch shortly with a set of requests. As we can't proceed until these requirements are met, your swift response will help prevent delays to publication.

Reviewer #1:
None
Response: thank you!

Reviewer #2 (Viviana I Perez):
In this revised manuscript the authors addressed all previous reviewers’ questions and concerns, which resulted in an strengthen manuscript.
Response: thank you!

Reviewer #3:
For the most part, the authors have satisfactorily addressed the majority of our concerns.
Response: thank you!

Reviewer #4:

Authors have satisfactorily addressed my comments.

However, authors have to revised carefully their data because there are still errors. I have not revised everything, but the few things I checked have errors. A few examples regarding Fig. S1D and the corresponding Table S1.1:

Response: We are very grateful to the reviewer for bringing these inconsistencies to our attention and helping to correct them in the latest version of the manuscript. We have addressed the issues listed below and a few other minor errors that were found upon re-checking of the text, data, and figures.

- LMNB1 in AVT-SEN-fibros: the figure says it goes up, however the

table says that it goes down

- LAMB1 on the contrary goes up in all three conditions. I wonder

if the authors wrongly select as UP in all conditions the protein

LMNB1, when it should have been LAMB1

Response: The reviewer is correct that this protein should have been LAMB1, rather than LMNB1. The reviewer previously pointed out that we had referred to Lamin-B1 (LMNB1) in the text and LAMB1 in the figure, and we had changed it to match. However, it turns out the figure was correct and the text was wrong. We have adjusted the figure back to LAMB1 and the text (line 179) to the correct corresponding protein, Laminin subunit beta-1 (LAMB1). We apologize for missing this in the previous round. We thoroughly checked everything to ensure that this is now all correct.

- HMGB1 in exosome of IR-SEN-fibros: the figure says it goes up,

the table says it goes down

- HMGB1, MMP2, TIMP1/2, IGFBP3/4/5/7: the figure says all go down,

the table says all go up

Response: We have corrected all arrows in the IR-exosomes to correctly correspond to the data in the supplementary table. We have also double checked that each other protein/condition/fraction in the table matches to the corresponding supplementary tables. Slight changes were made in the corresponding text to reflect the changes (lines 186-188).

Please, revise everything. I can understand a few errors, but these seem to me as too many.

Response: Again, we appreciate the reviewer catching the above mistakes and have appropriately read through the text and made minor adjustments to ensure consistency and accuracy between the text, figures and data tables. We have made a few additional adjustments in addition to the ones the reviewer has brought to our attention, including:

- The directions of CXCL8 and IGFBP3 in Fig S1 D were adjusted to accurately reflect the epithelial SASP. Slight changes were made in the corresponding text to reflect the changes (lines 180-184).
- Added text to Fig S1 legend (lines 1042-1044): “Red up-arrow = significantly increased (q-value < 0.05), blue down-arrow = significantly decreased (q-value < 0.05), hyphen (-) = not significant.”
- Filled in missing values in the “Absolute AVG Log2 Ratio” columns in Table S1.
- Removed CXCL8 from IR-specific signature (Fig 3A and S7 Table) and replaced with CLIPX (Fig 3A).
- Changed reference from Fig 2B to Fig 2C (lines 289, 296, and 404)
- Minor corrections to “IR (Epithelial)” column values in Table 1 (line 317), and corresponding text (line 308)
- Added text (line 404) “, in addition to other top biomarker candidates: MMP1, STC1, and GDF15 (Fig 7B-D)”
- Changed reference from S4 Fig to S5 Fig (line 260)
